# A Novel PPARγ Modulator Falcarindiol Mediates ER Stress-Mediated Apoptosis by Regulating NOX4 and Overcomes Radioresistance in Breast Cancer

**DOI:** 10.3390/antiox13121533

**Published:** 2024-12-14

**Authors:** Tae Woo Kim, Seong-Gyu Ko

**Affiliations:** 1 Department of Biopharmaceutical Engineering, Dongguk University-WISE, Gyeongju 38066, Republic of Korea; 2 Department of Preventive Medicine, College of Korean Medicine, Kyung Hee University, Seoul 02447, Republic of Korea

**Keywords:** falcarindiol, Nox4, ER stress, cell death, ROS

## Abstract

The extract of the rhizome of *Cnidium officinale* Makino has potential anti-cancer and anti-inflammatory effects in many diseases, such as cancer. However, the biological functions of falcarindiol (FAD) in breast cancer are not fully understood. This study proved the anti-inflammatory and anti-cancer effects of FAD in breast cancer. Breast cancer models confirmed that FAD reduces cell viability and decreases the tumor volume of xenograft mouse models in a dose-dependent manner. FAD mediated caspase-3-dependent apoptosis in MDA-MB-231 and MCF-7 cells, whereas Z-VAD-FMK in combination with FAD inhibited caspase-3-induced apoptosis. FAD mediates apoptosis through cytosolic reactive oxygen species (ROS) and calcium (Ca^2+^) production and ER stress signaling pathways. In addition, FAD combined with thapsigargin (TG) exerts a synergistic apoptotic cell death effect. In the loss-of-function experiments, PERK or CHOP ablation suppressed intracellular ROS and Ca^2+^ release and ER stress-induced apoptosis in FAD-treated breast cancer models. Since there is a relationship between ROS and NADPH Oxidase 4 (NOX4), Nox4 ablation blocked ER stress-mediated apoptotic cell death by inhibiting ROS release in FAD-induced breast cancer models. Radioresistant models, such as MCF-7R and MDA-MB-231R, were developed to address the cellular radioresistance in clinical radiotherapy. FAD combined with radiation (2 Gy) overcame radioresistance via the inhibition of the epithelial–mesenchymal transition (EMT) phenomenon, such as the upregulation of PPARγ, *VIM*, and *CDH2* and the downregulation of *CDH1*. Consequently, these results show that FAD may be a novel treatment as a breast cancer therapy.

## 1. Introduction

In females, breast cancer is frequently diagnosed and it ranked first in mortality and incidence worldwide in 2020 [1]. In the United States, every year, breast cancer-related death accounts for approximately 30% of all female cancer-related deaths [2]. Breast cancer therapy includes five therapeutic strategies: surgery, hormone therapy, chemotherapy, targeted therapies, and radiation therapy (radiotherapy). Most breast cancer therapies use a combination of these strategies [3]. Chemotherapy and radiotherapy are common therapeutic strategies for breast cancer patients; however, cancers frequently develop resistance to these strategies [4]. To overcome the resistance to breast cancer therapies, the development of new anti-cancer agents to potentially treat breast cancer is important.

Radiotherapy is a powerful therapeutic strategy used to treat various cancers [5]. Radiotherapy sensitizes cancer cells using high doses of radiation. Radiotherapy induces cancer cell death by causing damage to DNA and genetic changes [6]. However, radioresistance in cancer frequently induces many molecular mechanisms, including epithelial–mesenchymal transition (EMT), radiation-mediated DNA damage, and cell cycle dysregulation [7]. Therefore, a deeper understanding of the molecular mechanisms of radioresistance may aid in developing anti-cancer drugs and improve cancer therapies. Previous reports suggest that natural products and their derivatives, in combination with radiation, are potential tumor therapeutic strategies to overcome radioresistance and reduce radiation-induced side effects [8].

Falcarindiol (FAD) is a polyacetylene found in the roots of carrot family members that has anti-inflammatory, anti-cancer, and anti-bacterial activities [9,10,11]. Recent studies have reported that FAD derived from carrot roots has a potential anti-colon cancer effect by killing colon cancer cells, inhibiting tumor growth, and accumulating ubiquitinated proteins [12]. FAD combined with 5-fluorouracil (5-FU) exerts a synergistic anti-colon cancer effect by reducing cell viability. Additionally, FAD combined with cisplatin enhances chemosensitivity by inhibiting the STAT3-PTTG1 pathway in hepatocellular carcinoma [13]. Therefore, radiation combined with FAD may overcome radioresistance in breast cancer.

The endoplasmic reticulum (ER) plays a function in calcium (Ca^2+^) storage, ROS generation, and protein folding [14]. Accumulation of misfolded and unfolded proteins mediates ER stress by activating the unfolded protein response (UPR) transmembrane proteins, including inositol requiring enzyme 1α (IRE1α), PKR-like ER kinase (PERK), and activating transcription factor 6 (ATF6) [15,16]. Since there is a relationship between ER stress and oxidative stress, ER stress is induced by ROS-mediated signaling and ROS accumulation, leading to apoptosis and cell death [17,18]. Previous reports have shown that increased ROS generation induces the release of intracellular Ca^2+^ from the ER [19]. NADPH oxidases (Noxs) are localized to the ER, and the interaction between Noxs and ER is related to protein folding [20]. Nox-derived ROS generation is related to calcium release and is an important mechanism for ER-related functions, including ER stress-induced apoptotic cell death [21,22]. Nox4 regulates ER membrane-located enzyme sarcoendoplasmic reticulum calcium ATPase (SERCA), and the knockdown of Nox4 expression inhibits intracellular ROS and Ca^2+^ release, apoptotic cell death, and ER stress responses by blocking SERCA cys674 oxidation [23]. Thus, the intracellular ROS and Ca^2+^ relationship induces apoptotic cell death via ER stress in cancers [24]. In tumor environments, a better understanding of this relationship may improve the effect of anti-cancer therapies.

Therefore, this study aimed to identify a breast tumor therapeutic that overcomes radioresistance by activating Nox4 and ER stress-induced apoptotic cell death in FAD-treated breast cancer and radioresistant breast cancer cells.

## 2. Materials and Methods

### 2.1. Reagents

FAD (S3292) was acquired from Selleckchem (Houston, TX, USA). N-acetylcysteine (NAC), diphenyleneiodonium (DPI), thapsigargin (TG; Millipore, Bedford, MA, USA; T9033), and Z-VAD-FMK, lipopolysaccharide (LPS; L4391) were purchased from Sigma-Aldrich (St. Louis, MO, USA).

### 2.2. Cell Culture

MCF10A cells, a human breast cell line, were acquired from the American Type Culture Collection (ATCC, Manassas, VA, USA) and cultured in DMEM/F12 (Welgene, Gyeongsan, Republic of Korea) supplemented with 10% fetal bovine serum (FBS, Welgene, Gyeongsan, Republic of Korea), hydrocortisone (0.5 μg/mL, Sigma, Cream Ridge, NJ, USA), insulin (0.01 mg/mL, Sigma, Cream Ridge, NJ, USA), epithelial growth factor (EGF, 20 ng/mL, Invitrogen), 100 units/mL penicillin (Welgene, Gyeongsan, Republic of Korea), and 100 mg/mL streptomycin (Welgene, Gyeongsan, Republic of Korea). The human breast cancer cells MCF-7, HCC1419, T47D, HT20, SK-BR-3, and MDA-MB-231, and the pre-adipose mouse cell line 3T3L1, were purchased from the Korean Cell Line Bank (Cancer Research Center, Seoul, Republic of Korea) and cultured in DMEM (Welgene, Gyeongsan, Republic of Korea) supplemented with 10% FBS (Welgene, Gyeongsan, Republic of Korea), 100 units/mL penicillin (Welgene, Gyeongsan, Republic of Korea), and 100 mg/mL streptomycin (Welgene, Gyeongsan, Republic of Korea). All cells were maintained at 37 °C in a 5% CO_2_ incubator.

### 2.3. Cell Viability and Proliferation Assay

To confirm the efficacy of FAD on the viability of breast cancer cell lines at various doses and times, a WST-1 assay (Roche Applied Science, Indianapolis, IN, USA) was carried out. Breast cancer cells (1 × 10^4^ cells/well) were plated and maintained in a 96-well plate. The absorbance of each sample was monitored and investigated at 450 nm using a microplate reader (Molecular Devices, San Jose, CA, USA). The assay was carried out with regard to the manufacturer’s protocol.

### 2.4. Cytotoxicity LDH Assay

To identify the effect of FAD on cytotoxicity LDH in breast cancer cells at various doses and times, an LDH cytotoxicity assay (Abcam, Cambridge, MA, USA) was performed. Breast cancer cells (1 × 10^4^ cells/well) were plated and maintained in a 96-well plate. The assay was carried out with regard to the manufacturer’s protocol. The absorbance of each sample was monitored and investigated at 490 nm using a microplate reader (Molecular Devices, San Jose, CA, USA).

### 2.5. Caspase-3 Activity Assays

To identify the effect of FAD on the caspase-3 activity in breast cancer cells at many doses and times, a caspase-3 activity assay (Abcam, Cambridge, MA, USA) was performed. Breast cancer cells (1 × 10^4^ cells/well) were plated and maintained in a 96-well plate. The assay was carried out with regard to the manufacturer’s protocol. The absorbance of each sample was monitored and investigated at 490 nm using a microplate reader (Molecular Devices, San Jose, CA, USA).

### 2.6. Intracellular Ca^2+^ Assays

To confirm the efficacy of FAD on the generation of intracellular calcium in breast cancer cell lines at various doses and times, an intracellular Ca^2+^ assay (Abcam, Cambridge, MA, USA) was performed. Breast cancer cells (1 × 10^4^ cells/well) were plated and maintained in a 96-well plate. To quantify the intracellular Ca^2+^ levels, the fluorescence was monitored and investigated at 575 nm using a FilterMax F5 (Molecular Devices, San Jose, CA, USA) fluorescence microplate reader. This assay was carried out with regard to the manufacturer’s protocol.

### 2.7. Intracellular ROS Assays

To confirm the efficacy of FAD on ROS generation in breast cancer cell lines at various doses and times, an intracellular ROS assay (Abcam, Cambridge, MA, USA) was performed. Breast cancer cells (1 × 10^4^ cells/well) were plated and maintained in a 96-well plate. To quantify the intracellular ROS levels, the fluorescence was measured and analyzed at Ex/Em = 520 and 605 nm using a FilterMax F5 (Molecular Devices, San Jose, CA, USA) fluorescence microplate reader. The assay was performed following the manufacturer’s instructions.

### 2.8. Development of Radioresistant MDA-MB-231 and MCF-7 Cells

MDA-MB-231 and MCF-7 cells were seeded in 60 mm cell culture dishes. After 24 h, cells were exposed to radiation at the indicated dose of 4 Gy daily for 3 months to develop radioresistant MDA-MB-231 and MCF-7 cells. This course was reiterated, and then the radioresistant cells (MDA-MB-231R and MCF-7R) were developed.

### 2.9. Irradiation Experiment

MDA-MB-231, MCF-7, MDA-MB-231R, and MCF-7R cells were seeded in 60 mm dishes and incubated at 37 °C CO_2_ for 24 h. The cells were treated with radiation from a 137Cs source (Atomic Energy of Canada, Ltd., Mississauga, ON, Canada). Developed MDA-MB-231R and MCF-7R cells were treatd to 4 Gy for 3 months and maintained in a growth medium.

### 2.10. Colony Formation Assay

MDA-MB-231, MCF-7, MDA-MB-231R, and MCF-7R cells were seeded in 60 mm dishes with growth medium. Cells were maintained for ten days to form colonies. And the colonies were stained using crystal violet (0.5%, Sigma, Cream Ridge, NJ, USA). This assay was carried out with regard to the manufacturer’s protocol.

### 2.11. Transfection

siRNAs for PERK (Santa Cruz Biotechnology, Dallas, TX, USA), Nox4 (Santa Cruz Biotechnology, Dallas, TX, USA), and CHOP (Bioneer, Daejeon, Republic of Korea), and PPARγ shRNA particles (Santa Cruz Biotechnology, Dallas, TX, USA), were purchased. MDA-MB-231 and MCF-7 cells were plated in a six-well plate and transfected with siRNAs (30 nmol/mL) using Lipofectamine 2000 (ThermoFischer Scientific, Waltham, MA, USA). The cells were then treated with PPARγ shRNA particles (20 μL) with regard to the manufacturer’s protocol. To perform the luciferase reporter assay, breast cancer cells MDA-MB-231 and MCF-7 cells and 3T3-L1 murine adipocytes were co-transfected with the pGL3 PPRE-luciferase reporter vector (1.5 µg, Promega, Madison, WI, USA) or the pGL3 luciferase reporter vectors (1.5 µg, Promega, Madison, WI, USA) using a luciferase assay system (Promega, Madison, WI, USA). Following the manufacturer’s protocol, the luciferase activity at 550 nm was measured from each well using FilterMax F5.

### 2.12. RNA and Protein Purification Assay

Cells were seeded in a 100 mm cell culture dish. Total RNA from the MDA-MB-231 and MCF-7 cells were purified with Trizol (Invitrogen) with regard to the manufacturer’s protocol. Protein from the cell lysates was extracted using RIPA buffer (ThermoFischer Scientific, Waltham, MA, USA).

### 2.13. RT-qPCR

For the RT-qPCR analysis, triplicate reactions were performed. The primer sequences were as follows: *GRP78* (i.e., *HSPA5*) 5′-TCAGCCCACCGTAACAAT-3′ (sense) and 5′-CAAACTTCTCGGCGTCAT-3′ (antisense), *ATF4* (5′-AAGCCTAGGTCTCTTAGATG-3′ (sense) and 5′- TTCCAGGTCATCTATACCCA-3′ (antisense), *CHOP* (i.e., *DDIT3*) (5′- ATGAGGACCTGCAAGAGGTCC-3′ (sense) and 5′-TCCTCCTCAGTCAGCCAAGC-3′ (antisense), *CDH1* 5′- GAACGCATTGCCACATACAC-3′ (sense) and 5′-GAATTCGGGCTTGTTGTCAT-3′ (antisense), *CDH2* 5′-GGCATACACCATG CCATCTT-3′ (sense) and 5′-GTGCATGAAGGACAGCCTCT-3′ (antisense), and *VIM* 5′-GAGAACTTTGCCGTTGAAGC-3′ (sense) and 5′-GCTTCCTGTAGGTGGCAATC-3′ (antisense). The reaction was performed using a Roche LightCycler 96 System (Roche, Indianapolis, IN, USA). Extracted RNA were normalized to *ACTB* using the primer 5′-AAGGCCAAC CGCGAGAAGAT-3′ (sense) and 5′-TGATGACCTGGCCGTCAGG-3′ (antisense). The relative expression value for the gene expression was analyzed with the 2^−ΔΔCt^ method.

### 2.14. Western Blot Analyses

A Western blotting assay was carried out to identify the expression level of the various proteins. The membranes were blocked using skim milk (5%) and maintained using target primary antibodies. The target primary antibodies used were GRP78 (Santa Cruz Biotechnology, 1:1000, sc-166490), eIF2α (Santa Cruz Biotechnology, 1:1000, sc-133132), β-actin (Santa Cruz Biotechnology, 1:1000, sc-47778), p-eIF2α (Ser51) (Cell Signaling, 1:1000, #3398), Nox4 (Proteintech, 1:1000, 14347-1-AP), p-PERK(Thr980) (Cell Signaling, 1:1000, #3179), cleaved caspase-9 (Cell Signaling, 1:1000, #20750), PERK (Cell Signaling, 1:1000, #5683), CHOP (Cell Signaling, 1:1000, #2895), cleaved caspase-3 (Cell Signaling, 1:1000, #9664), PPARγ (Proteintech), and ATF4 (Cell Signaling, 1:1000, #11815). The membranes were then maintained with the following HRP-conjugated secondary antibodies: m-IgGK BP-HRP-linked antibody (Santa Cruz Biotechnology, 1:5000, sc-2357) or anti-mouse anti-rabbit IgG HRP-linked antibody (Santa Cruz Biotechnology, 1:5000, sc-516102). The membranes were analyzed with an ECL (Millipore, Bedford, MA, USA).

### 2.15. Animal Testing and Experimentation

Athymic BALB/c nude mice (female, five-week-old) were acquired from OrientBio, Inc. (Daejeon, Republic of Korea). The mice were maintained in a specific pathogen-free (SPF) facility for one week and fed a diet of NIH-7 open formula. The mice were divided randomly into three groups. All animal testing and experimentations were carried out with regard to the guidelines of the NIH and the Kyung-Hee University Animal Care and Use Committee. For the animal studies using xenograft mice, breast cancer cells (MDA-MB-231(1 × 10^7^)) were mixed with PBS and injected subcutaneously (sc) into the right dorsal flank of the mice. Since the tumor volumes arrived approximately 200 mm^3^, the mice were randomly divided (n = 10 mice/group). FAD (10 or 20 mg/kg) was injected intraperitoneally (ip) twice weekly. Tumor volume was monitored and estimated according to the following formula: (*L* × *W*^2^)/2 (mm^3^). To identify the FAD-induced anti-inflammatory effect, mice were randomly divided into three groups (PBS, LPS, and LPS + FAD). Previous studies have shown that a dose of 20 mg/kg LPS via intraperitoneal injection induces inflammation. FAD was injected intraperitoneally following the LPS injection and the mice were evaluated for 12 days, and blood and tissue samples were collected. Moreover, the survival rate was monitored and analyzed for 2–12 days after the LPS injection.

### 2.16. Cytokine Measurements

Raw264.7 and J774.1 cells (1 × 10^4^ cells/well) were plated into a 96-well plate. To investigate the cytokines produced from Raw264.7 and J774.1 cells, these cells were treated with LPS (1 μg/mL) and FAD (0, 25, 50, and 100 μM; 24 h) and then an enzyme-linked immunosorbent assay (ELISA) was performed. The expression levels of the cytokines, including IL-1β, IL-6, and TNF-α, were monitored and investigated using the following ELISA kits: IL-1β (DY-401; R&D Systems), IL-6 (DY-406; R&D Systems), and TNF-α (DY-410; R&D Systems). This assay was carried out with regard to the manufacturer’s protocol.

### 2.17. Statistical Analysis

The results were proved in at least three independent tests. The statistical significance was evaluated using Student’s *t*-test (* *p <* 0.05; n.s, not significant).

## 3. Results

### 3.1. FAD Regulates PPARγ Activity in 3T3L1 and Breast Cancer Cells

**Figure 1:**
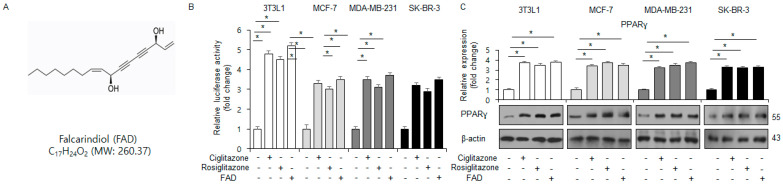
Identification of falcarindiol as a PPARγ activator. (**A**) The chemical structure of falcarindiol. (**B**,**C**) The luciferase activity of the PPAR response element reporter gene and expression levels of mRNA and protein in falcarindiol- (7.5 µM), ciglitazone- (10 µM), and rosiglitazone- (20 µM) treated 3T3L1, MCF-7, MDA-MB-231, and SK-BR-3 cells. * = *p* < 0.05.

Figure 1A shows the chemical structure of FAD. A PPRE luciferase-driven reporter assay, an RT-qPCR assay, and a Western blotting assay showed that FAD (7.5 µM) increased PPRE luciferase activity on PPARγ promoter and PPARγ expression in PPARγ ligand ciglitazone-, rosiglitazone-, and FAD-treated 3T3L1, SK-BR-3, MDA-MB-231, and MCF-7 cells (Figure 1B,C).

### 3.2. FAD Reduces LPS-Induced Pro-Inflammatory Cytokines in Macrophages

**Figure 2:**
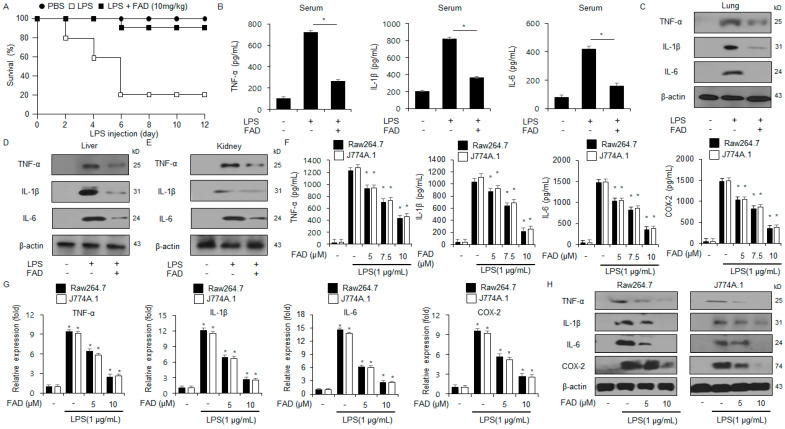
Effects of falcarindiol on the mRNA and protein expressions of inflammatory response markers in LPS-treated macrophages. (**A**) C57BL/6 mice were injected intraperitoneally (i.p.) with LPS (20 mg/kg) and LPS combined with FAD (10 mg/kg). After the injection, the survival rate of the LPS alone and LPS combined with FAD groups (*n* = 10/group) was analyzed daily for 12 days. (**B**–**E**) Protein expression levels of TNF-α, IL-6, and IL-1β in the serum and tissues such as lung, liver, and kidney in the LPS-mediated mouse models using ELISA and Western blot assays. (**F**–**H**) mRNA and protein levels of TNF-α, IL-6, and IL-1β in LPS (1 µg/mL)-treated Raw264.7 and J774.1 cells with and without FAD (0, 5, 7.5, and 10 µM, 24 h) measured by an ELISA, Western blot, and RT-qPCR. β-actin was used to normalize the relative mRNA and protein levels. * = *p* < 0.05. These experiments were repeated three times.

To demonstrate the inflammatory effects of FAD, in vivo experiments using an LPS-mediated mouse model were performed. Compared with the LPS group, the LPS and FAD (10 mg/kg) group had an increase in the survival rate of approximately four-fold (Figure 2A). To further identify the anti-inflammatory effects of FAD on LPS-induced inflammation, the gene expression degrees of inflammation markers, including IL-1β, IL-6, and TNF-α, were investigated using mouse tissues, including lungs, liver, and kidney, and serum samples. The FAD treatment downregulated the levels of IL-1β, IL-6, and TNF-α in the tissue and serum from the LPS-induced mouse models (Figure 2B–E). To identify whether FAD modulates the anti-inflammatory effects in LPS-treated Raw264.7 and J774.1 cells, an RT-qPCR, Western blotting assay, and ELISA were performed. First, an ELISA assay was performed to identify whether FAD regulates the expression of IL-1β, IL-6, and TNF-α in LPS-treated J774.1 and Raw264.7 cells. After pre-treating the Raw 264.7 and J774.1 cells with LPS, the treatment of FAD reduced the expressions of IL-6, IL-1β, and TNF-α (Figure 2F). In addition, the mRNA levels of COX-2, IL-1β, IL-6, and TNF-α were increased in the Raw264.7 and J774.1 cells treated with LPS. Based on these results, the mRNA levels of these cytokines were decreased in the FAD-induced J774.1 and Raw264.7 cells (Figure 2G). The Western blot analysis showed that FAD decreased the protein levels of COX-2, IL-1β, IL-6, and TNF-α in the LPS-treated J774.1 and Raw264.7 cells (Figure 2H). These results suggest that the cytokines, including IL-6, IL-1β, and TNF-α, are suppressed by FAD in LPS-induced Raw264.7 and J774.1 cells.

### 3.3. FAD Mediates Anti-Cancer Efficacy in Breast Cancer Cells

**Figure 3:**
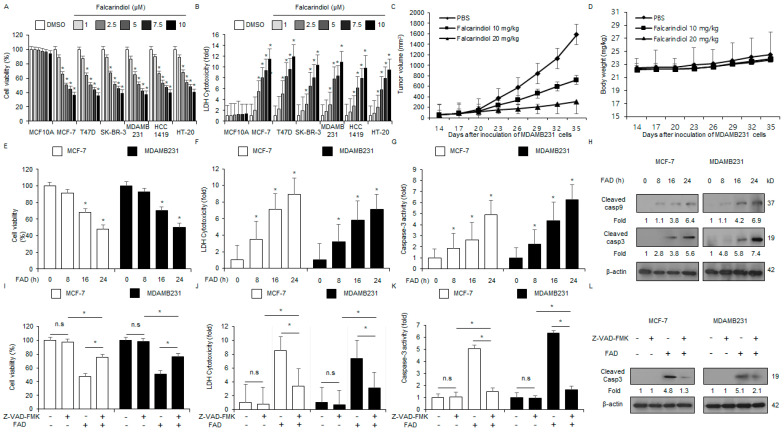
Anti-breast cancer effect of falcarindiol in vivo and in vitro. (**A**,**B**) LDH cytotoxicity and cell viability were measured using WST-1 and LDH cytotoxicity assays at the indicated concentrations (0, 1, 2.5, 5, 7.5, and 10 µM, 24 h) in FAD-mediated normal breast and cancer cells (MCF10A, HT-20, SK-BR-3, T47D, HCC1419, MDA-MB-231, and MCF-7). (**C**,**D**) MDA-MB-231 cells (4 × 10^6^) were injected (sc) into the right dorsal flank of nude mice (n = 10/group). FAD (10 and 20 mg/kg) was administered (ip) twice weekly. The body weights of the MDA-MB-231 tumor-xenograft mice were determined twice a week. (**E**–**H**) In the MCF-7 and MDA-MB-231 cells, FAD treatments were administered in a time-dependent manner (0, 8, 16, and 24 h; 7.5 µM) and various assays, including caspase-3 activity, LDH assay, and WST-1 assays, were conducted. The Western blot assay for cleaved caspase-9 and caspase-3 was performed in a time-dependent manner in FAD-treated MDA-MB-231 and MCF-7 cells; * *p* < 0.05. β-actin was used as the protein loading control. (**I**–**L**) MDA-MB-231 and MCF-7 cells were pre-treated with Z-VAD-FMK (50 μM) for 4 h and then treated with FAD (7.5 µM, 24 h). Various biological assays, including caspase-3 activity, LDH assay, and WST-1, were performed (* *p* < 0.05, n.s = no significance.). A Western blot assay was performed to identify the caspase-3 cleavage using a cleaved caspase-3 antibody. β-actin was used as the loading control.

To confirm the anti-cancer efficacy of FAD in breast cancer cell lines, including HT-20, MDA-MB-231, SK-BR-3, MCF-7, T47D, and HCC1419, and normal breast cell lines (MCF10A), these were treated with various amounts of FAD (1, 2.5, 5, 7.5, and 10 µM). LDH cytotoxicity and WST-1 assays were performed (Figure 3A,B). To more fully investigate the anti-tumor efficacy of FAD, a xenograft assay was performed. A xenograft nude mouse model was established using MDA-MB-231 cells and FAD injections of 10 and 20 mg/kg. The tumor volumes were analyzed and compared (Figure 3C). There was no observed effect on the change in body weight following the FAD treatment (Figure 3D). To confirm the tumor suppressive efficacy of FAD, the cells were treated with 7.5 µM FAD at the indicated times (0, 8, 16, and 24 h) and then cytotoxicity LDH, WST-1, and colorimetric caspase-3 activity assays were carried out. FAD treatments induce a time-dependent inhibition of cell viability and a time-dependent increase in colorimetric caspase-3 activity and LDH cytotoxicity (Figure 3E–G). Western blot assays indicated that FAD mediates the cleavage of caspase-3 and caspase-9 at the indicated times (Figure 3H). To further identify if FAD induces caspase-caused apoptosis in breast cancer, a pharmacological study was performed using the pan-caspase inhibitor Z-VAD-fmk in MDA-MB-231 and MCF-7 cell lines. Z-VAD-fmk (50 μM) did not affect caspase-3 activity, cell viability, and cytotoxicity LDH; however, FAD (7.5 µM) increased the caspase-3 activity and LDH cytotoxicity and reduced cell viability. Combined with FAD (7.5 µM), Z-VAD-fmk (50 μM) blocked the significant increase in caspase-3 activity and cytotoxicity LDH and the decrease in cell viability (Figure 3I–K). In addition, the Western blotting assays showed that the combined Z-VAD-fmk and FAD downregulated the expression levels of caspase-3 cleavage compared with the FAD treatment (Figure 3L). These findings indicate that FAD mediates anti-tumor efficacy by activating caspase-caused apoptosis in breast cancer.

### 3.4. FAD Induces Apoptotic Cell Death via ER Stress Response in Breast Cancer

**Figure 4:**
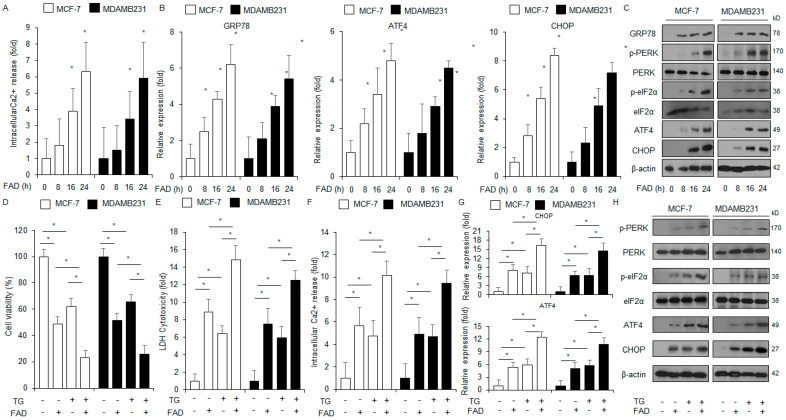
Falcarindiol induces ER stress-mediated apoptotic cell death via intracellular Ca^2+^ release. (**A**) MDA-MB-231 and MCF-7 cells were treated with FAD in a time-dependent manner (0, 8, 16, and 24 h; 7.5 µM). An intracellular Ca^2+^ assay was performed (* *p* < 0.05). (**B**) mRNA expression levels of *ATF4*, *CHOP*, and *GRP78* were assessed using an RT-qPCR. *ACTB* was used as the loading control. (**C**) MDA-MB-231 and MCF-7 cells were treated with FAD (0, 8, 16, and 24 h; 7.5 µM), and then the ER stress-related signaling pathway was assessed via proteins such as ATF4, CHOP, GRP78, p-eIF2α, and p-PERK using a Western blot assay. β-actin was used as the protein loading control. (**D**–**F**) MDA-MB-231 and MCF-7 cells were treated with thapsigargin (TG; 3 μM, 24 h) and FAD (7.5 µM, 24 h). Various assays, including intracellular Ca^2+^ assays, cell viability, and LDH cytotoxicity, were performed (* *p* < 0.05). (**G**,**H**) An RT-qPCR was performed to identify the mRNA levels of *CHOP* and *ATF4* and a Western blot assay was performed to confirm the protein expression levels of CHOP and ATF4 and the phosphorylation levels of eIF2α and PERK in FAD (7.5 µM, 24 h) and thapsigargin (TG; 3 μM, 24 h)-treated MDA-MB-231 and MCF-7 cells. β-actin was used as the loading control.

Calcium (Ca^2+^) signaling plays a role in regulating cell homeostasis and processes, including cell survival, cell death, immune response, and migration, in various cancer cell types [25]. The ER is an important location for Ca^2+^ storage [26]. Prolonged or excessive intracellular Ca^2+^ and ROS release are potential drivers of cancer growth, and the interaction between Ca^2+^ and ROS is a powerful tumor therapeutic strategy [27]. Previous studies have indicated that increased intracellular Ca^2+^ and ROS release mediates apoptotic cell death through the unfolded-protein-induced ER stress response [28]. To confirm whether FAD increases intracellular Ca^2+^ in breast cancer cell lines at the indicated times, an intracellular Ca^2+^ assay was performed. There was increased production of intracellular Ca^2+^ in the cells treated with FAD (Figure 4A). Furthermore, an RT-qPCR and a Western blot assay were performed in MDA-MB-231 and MCF-7 cells. FAD upregulated the mRNA levels of *CHOP*, *GRP78*, and *ATF4* and the protein levels of p-PERK, p-eIF2α, CHOP, GRP78, and ATF4 (Figure 4B,C). Prolonged or excessive ER stress response has an anticancer effect via activating apoptotic cell death [29]. To confirm whether the ER stress response was regulated with FAD-induced apoptosis, MDA-MB-231 and MCF-7 cell lines were co-treated with FAD and thapsigargin (TG, ER stress inducer), and then WST-1, LDH cytotoxicity, and intracellular Ca^2+^ assays were carried out. FAD, in combination with the TG treatment, mediated a synergistic inhibition in cell viability and an enhancement in cytotoxicity LDH and intracellular Ca^2+^ generation (Figure 4D–F). To further identify if FAD combined with TG regulates ER stress markers and signaling pathways in MDA-MB-231 and MCF-7 cell lines, a real-time RT-PCR and a Western blotting assay were performed. The combined treatment of FAD and TG mediated the phosphorylation of eIF2α and PERK and upregulated CHOP and ATF4 (Figure 4G,H).

### 3.5. Inhibition of PERK or CHOP Suppresses FAD-Induced Apoptosis in Breast Cancer

**Figure 5:**
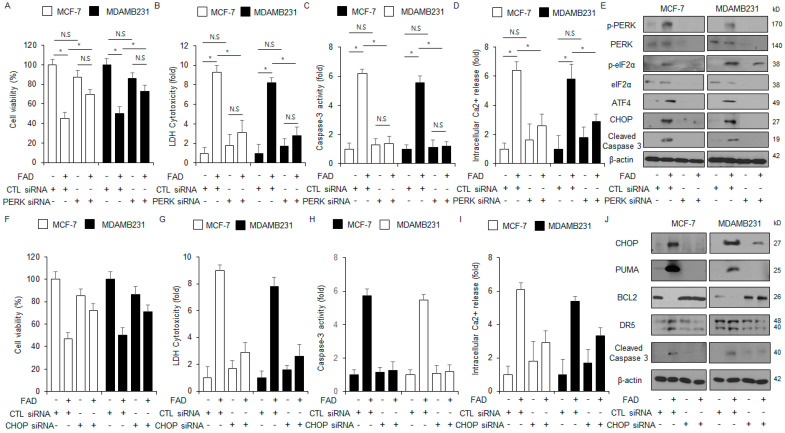
Targeting PERK or CHOP blocks FAD-mediated apoptotic cell death in breast cancer. (**A**–**E**) PERK siRNA was transfected into MDA-MB-231 and MCF-7 cells and then treated with FAD (7.5 µM, 24 h). Various assays, including intracellular Ca^2+^ activity, WST-1, caspase-3 activity, and LDH cytotoxicity, were conducted (* *p* < 0.05, N.S = no significance). A Western blot assay was carried out to identify the phosphorylation levels of eIF2α and PERK and the protein expression levels of cleaved caspase-3, CHOP, and ATF4 in FAD (7.5 µM, 24 h)-treated MDA-MB-231 and MCF-7 cells. β-actin was used as the loading control. (**F**–**J**) CHOP siRNA was transfected into MDA-MB-231 and MCF-7 cells and then treated with FAD (7.5 µM, 24 h). Various assays, including intracellular Ca^2+^ activity, WST-1, caspase-3 activity, and LDH cytotoxicity, were conducted (* *p* < 0.05). A Western blot assay was performed to identify the protein expression levels of PUMA, BCL2, cleaved caspase-3, CHOP, and DR5 in FAD (7.5 µM, 24 h)-treated MDA-MB-231 and MCF-7 cells. β-actin was used as the loading control.

To examine if PERK knockdown modulates the FAD-induced apoptosis in MDA-MB-231 and MCF-7 cell lines, PERK-specific siRNAs were transfected into cells, and then various assays, including cytotoxicity LDH, WST-1, colorimetric caspase-3 activity, cytosolic Ca^2+^ activity, and Western blots, were carried out. These findings suggest that PERK knockdown suppresses the inhibition of cell viability and the enhancement in intracellular Ca^2+^ activity, colorimetric caspase-3 activity, and LDH cytotoxicity (Figure 5A–D). In addition, the Western blot assays showed that the inhibition of PERK mediates the dephosphorylation of p-eIF2α and p-PERK and the downregulation of cleaved caspase-3, ATF4, and CHOP in FAD-treated MDA-MB-231 and MCF-7 cells compared with the control (Figure 5E). To further identify whether the inhibition of CHOP modulates the FAD-induced apoptotic cell death, CHOP knockdown experiments using specific siRNAs were performed using FAD-treated MDA-MB-231 cells, and then various assays, including WST-1, LDH cytotoxicity, caspase-3, and intracellular Ca^2+^ activity, were performed. CHOP knockdown inhibited the suppression of cell viability and the enhancement in intracellular Ca^2+^ activity, colorimetric caspase-3 activity, and LDH cytotoxicity (Figure 5F–I). The Western blot assays demonstrated that the CHOP knockdown inhibited the downregulation of BCL2 and the enhancement of DR5, PUMA, CHOP, and caspase-3 cleavage compared with the control cells (Figure 5J). These findings suggest that targeting the ER stress pathway regulates apoptotic cell death in FAD-mediated breast cancer cells.

### 3.6. Loss of Nox4 Function Inhibits FAD-Induced Apoptosis in Breast Cancer

**Figure 6:**
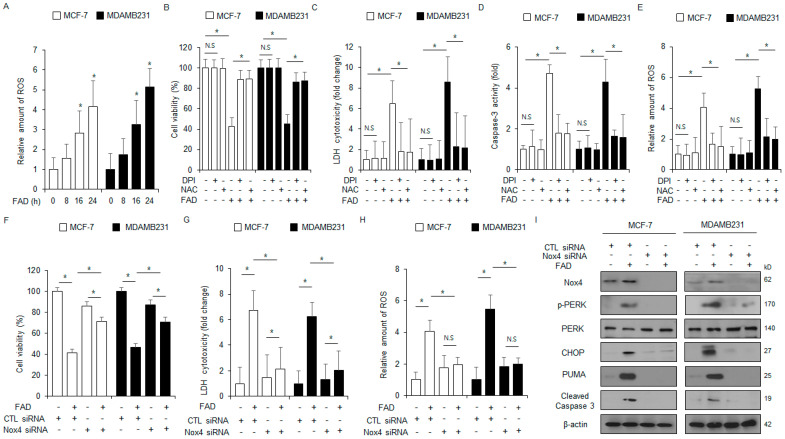
Targeting Nox4 blocks ROS-mediated ER stress and apoptosis in falcarindiol-treated breast cancer. (**A**) MDA-MB-231 and MCF-7 cells were treated with FAD (7.5 μΜ) in a time-dependent manner and an intracellular ROS activity assay using DCFDA dye was conducted (* *p* < 0.05). (**B**–**E**) MDA-MB-231 and MCF-7 cells were treated with FAD (7.5 μM, 24 h), DPI (1 μM, 24 h), and NAC (100 μM, 24 h) and then various assays, including caspase-3 activity, intracellular ROS production, LDH cytotoxicity, and WST-1, were performed (* *p* < 0.05). (**F**–**I**) NOX4 siRNA was transfected into MDA-MB-231 and MCF-7 cells and then treated with FAD (7.5 µM, 24 h). Various assays, including intracellular ROS activity, WST-1, and LDH cytotoxicity, were carried out (* *p* < 0.05, N.S = no significance). A Western blot assay was conducted to identify the protein expression levels of the PUMA, NOX4, cleaved caspase-3, CHOP, PERK, and p-PERK in FAD (7.5 µM, 24 h)-treated MDA-MB-231 and MCF-7 cells. β-actin was used as the loading control.

To verify if the FAD regulates intracellular ROS generation in breast cancer, an intracellular ROS assay was carried out. The FAD increased the ROS production at the indicated times (Figure 6A). To further examine whether the FAD treatment regulates intracellular ROS release, MDA-MB-231 and MCF-7 cell lines were co-treated with the ROS scavenger NAC or DPI and FAD, and then various assays, including intracellular ROS, cytotoxicity LDH, WST-1, and colorimetric caspase-3 activity, were performed. FAD, in combination with DPI or NAC, blocked the inhibition in cell viability and the enhancement in intracellular ROS production, caspase-3 activity, and LDH to a greater extent than the FAD alone (Figure 6B–E). To confirm whether FAD-induced intracellular ROS is related to Nox4 expression, Nox4 targeting siRNAs were transfected into MDA-MB-231 and MCF-7 cell lines, and the cells were treated with FAD. Various assays, including LDH cytotoxicity, intracellular ROS, WST-1, and Western blots, were performed. The inhibition of Nox4 resulted in higher cell viability and lower cytotoxicity LDH and intracellular ROS generation in the FAD-induced MDA-MB-231 and MCF-7 cells compared with the control cells (Figure 6F–H). The Western blot analyses demonstrated that the inhibition of Nox4 decreased Nox4, CHOP, caspase-3 cleavage, PUMA, and p-PERK levels in FAD-treated MCF-7 and MDA-MB-231 cells to a greater extent than in the control cells (Figure 6I). These findings suggest that Nox4 is related to the FAD-induced intracellular ROS production and regulates FAD-mediated apoptotic cell death in breast cancer cells.

### 3.7. Combination of FAD and Radiation Overcomes Radioresistance in Radio-Resistant Breast Cancer Models

**Figure 7:**
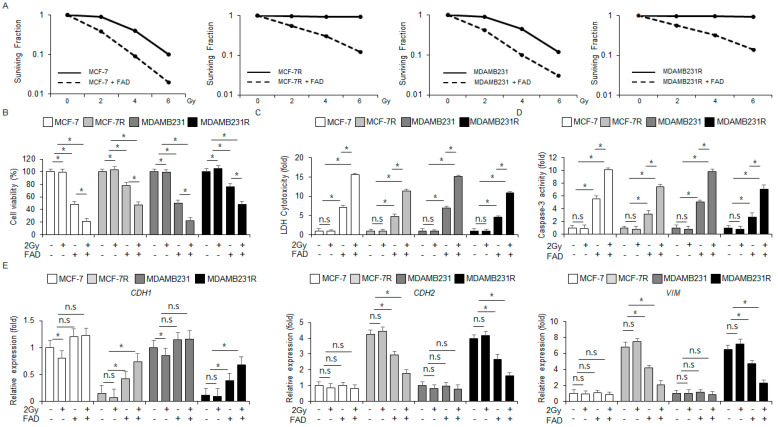
Falcarindiol combined with radiation overcomes radioresistance in radioresistant breast cancer cells. (**A**) A colony formation assay was conducted at the indicated concentrations (0, 2, 4, and 6 Gy) in FAD and radiation-co-treated MDA-MB-231, MCF-7, MDA-MB-231R, and MCF-7R cells. The survival fraction was measured (* *p* < 0.05, n.s = no significance). (**B**–**D**) MDA-MB-231, MCF-7, MDA-MB-231R, and MCF-7R cells were treated with FAD (7.5 µM, 24 h) combined with radiation (2 Gy, 24 h) and then various assays, including caspase-3 activity, WST-1 assay, and LDH cytotoxicity, were conducted (* *p* < 0.05, n.s = no significance). (**E**) An RT-qPCR was conducted to identify the mRNA levels of *VIM*, *CDH2*, and *CDH1* in MDA-MB-231, MCF-7, MDA-MB-231R, and MCF-7R cells treated with FAD (7.5 µM, 24 h) combined with radiation (2 Gy, 24 h) (* *p* < 0.05, n.s = no significance). *ACTB* was used as the mRNA loading control.

Radiation therapy is the most common treatment for breast cancer; however, breast cancer sufferers frequently acquire radioresistance [30]. To test if FAD overcomes radioresistance in MDA-MB-231R and MCF-7R, colony formation assays were performed. The FAD treatment lowered the surviving fraction values at the various radiations (2, 4, and 6 Gy) in MDA-MB-231R, MDA-MB-231, MCF-7, and MCF-7R cells to a greater extent than the control samples (Figure 7A). In the MCF-7 and MDA-MB-231 cell lines, FAD decreased cell viability and enhanced colorimetric caspase-3 activity and cytotoxicity LDH. Combined with radiation (2 Gy), FAD decreased cell viability and enhanced colorimetric caspase-3 activity and cytotoxicity LDH even more than the FAD treatment alone. Radiation (2 Gy) alone did not influence cytotoxicity LDH, cell viability, or colorimetric caspase-3 activity (Figure 7B–D). In the MCF-7R and MDA-MB-231R cell lines, the FAD decreased cell viability and increased LDH and colorimetric caspase-3 activity. Combined with radiation (2 Gy), FAD decreased cell viability and enhanced cytotoxicity LDH and colorimetric caspase-3 activity even more than the FAD treatment alone. Radiation (2 Gy) alone had no effect on cytotoxicity LDH, cell viability, and colorimetric caspase-3 activity (Figure 7B–D). To confirm if the combination of radiation (2 Gy) and FAD regulates the EMT process in MCF-7R and MDA-MB-231R, an RT-qPCR was performed. The RT-qPCR showed that FAD, or the combination of radiation (2 Gy) and FAD, reduced the levels of mesenchymal markers, including *VIM* and *CDH2*, whereas they enhanced the levels of epithelial cell marker *CDH1* in MCF-7R and MDA-MB-231R cell lines. In the MCF-7 and MDA-MB-231 cells, the expression levels of EMT markers were not influenced significantly (Figure 7E). These findings indicate that, combined with radiation, FAD could be a powerful tumor therapy by overcoming radioresistance.

### 3.8. PPARγ Knockdown Inhibits ER Stress-Mediated Apoptosis in FAD-Treated MCF-7R and MDA-MB-231R Cells

**Figure 8:**
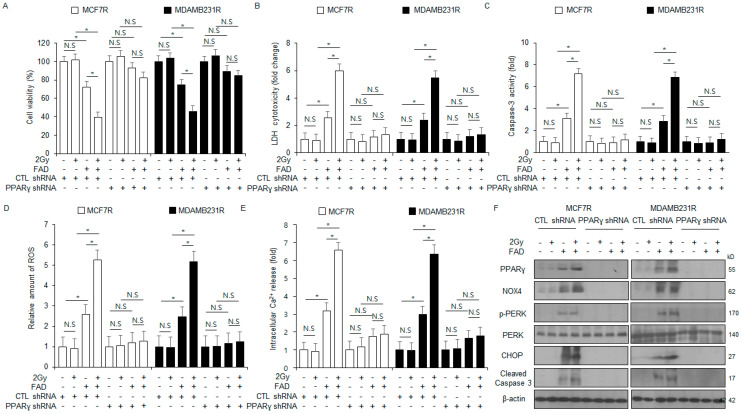
PPARγ knockdown blocks FAD combined with radiation-mediated apoptotic cell death in radioresistant MCF-7R and MDA-MB-231R cells. (**A**–**F**) PPARγ shRNA particles were transfected into MCF-7R and MDA-MB-231R cells, and stable PPARγ knockdown MCF-7R and MDA-MB-231R cells were established. After treating the PPARγ knockdown stable MCF-7R and MDA-MB-231R cells with a treatment of FAD (7.5 µM, 24 h) combined with radiation (2 Gy, 24 h), various assays, including LDH cytotoxicity, intracellular Ca^2+^, ROS activity, caspase-3 activity, WST-1, and Western blots, were conducted. A Western blot analysis was performed to determine the expression levels of NOX4, PERK, PPARγ, p-PERK, cleaved caspase-3, and CHOP (* *p* < 0.05, N.S = no significance). β-actin was used as the protein loading control.

To confirm if the combination of FAD and radiation (2 Gy) mediates apoptosis by regulating PPARγ, PPARγ shRNA was transfected into MCF-7R and MDA-MB-231R cells and stable PPARγ-knockdown cells were developed. These cells were exposed to FAD and/or radiation (2 Gy), and then various assays, including cytotoxicity LDH, WST-1, intracellular ROS and Ca^2+^ release, colorimetric caspase-3 activity, and Western blots, were carried out. In the control shRNA-transfected MCF-7R and MDA-MB-231R cell lines, the FAD inhibited cell viability and enhanced colorimetric caspase-3 activity, cytotoxicity LDH, and intracellular ROS and Ca^2+^ generation. The combination of FAD and radiation (2 Gy) decreased cell viability and enhanced colorimetric caspase-3 activity, cytotoxicity LDH, and intracellular ROS and Ca^2+^ release. Radiation (2 Gy) alone had no effect. In contrast, FAD or radiation or FAD + radiation had no effects in the stable PPARγ-knockdown MCF-7R and MDA-MB-231R cells (Figure 8A–E). The Western blotting assay demonstrated that FAD increased the expressions of CHOP, PPARγ, p-PERK, NOX4, and cleaved caspase-3; however, radiation alone did not change the expression. Compared with FAD alone, FAD combined with radiation (2 Gy) upregulated the levels of PPARγ, CHOP, p-PERK, NOX4, and caspase-3 cleavage (Figure 8F). In the stable PPARγ-knockdown MCF-7R and MDA-MB-231R cells, only FAD downregulated the expression of PPARγ, whereas radiation (2 Gy) or FAD combined with radiation (2 Gy) had almost no effects. These results suggest that FAD-induced PPARγ activation mediates apoptotic cell death via the relationship between oxidants and ER stress response and has the potential to overcome radioresistance in breast cancer.

## 4. Discussion

Previous reports have indicated that natural compounds have potential functions in cancer therapies [31,32]. In cancer, oxidative stress induces higher intracellular ROS and Ca^2+^ release-dependent apoptotic cell death. Targeting antioxidants or oxidants may be a powerful cancer therapy strategy [33,34]. Based on the findings of this study, the natural compound FAD is a powerful anti-cancer drug that can overcome radioresistance in breast cancer radiotherapy. The anti-cancer effect-related signaling pathway was studied in FAD-treated breast cancer cells. Apoptosis is a programmed cell death and is an important cellular target of anticancer therapies [35]. FAD induces caspase-dependent apoptotic cell death, and the cell-permeable pan-caspase inhibitor Z-VAD-fmk combined with FAD suppresses apoptosis in breast cancer cells. Excessive or prolonged ER stress response mediates apoptosis through the phosphorylation of the UPR transmembrane sensor PERK and the induction of CHOP [36]. FAD also induces the ER stress response through the generation of intracellular ROS and Ca^2+^ and mediates apoptotic cell death through the induction of the PERK-eIF2α-ATF4-CHOP axis. Targeting PERK or CHOP suppressed the FAD-mediated apoptosis, whereas the ER stress inducer TG mediated apoptosis in the FAD-treated breast cancer cells. The induction of Nox4 mediates apoptosis by producing ROS and by activating ER stress. FAD-mediated NOX4 activity and NOX4 expression induced ROS and intracellular Ca^2+^ production, apoptosis, and the ER stress response in breast cancer cell lines. However, the inhibition of Nox4 and the treatment with the ROS inhibitor DPI or NAC suppressed intracellular Ca^2+^ and ROS production, apoptosis, and UPR response in FAD-treated breast cancer cells. Radiotherapy is an important tumor therapeutic strategy; however, cancers frequently develop radioresistance [37]. Novel combination strategies to overcome radioresistance may contribute to effective therapies for cancer patients. FAD combined with radiation overcame radioresistance via the regulation of the EMT process in MCF-7R and MDA-MB-231R. Natural compounds have a powerful curative effect in cancer therapies with fewer adverse effects [38,39]. Additionally, natural compounds combined with traditional strategies increase the anti-cancer therapy effects via the enhancement of synergistic effects and the reduction of chemoresistance and side effects, resulting in promising novel tumor therapeutic strategies for cancer patients [40].

The natural compound curcumin, in combination with fluorouracil, induces apoptosis via the downregulation of BCL2 and the upregulation of cleaved caspase-9 and caspase-3 in liver cancer cells [41]. The natural compound cinnamaldehyde induces ER stress-caused apoptosis through the PERK axis in gastric cancer cell lines, and cinnamaldehyde combined with the ER stress inducer TG mediates apoptotic cell death [42]. The natural product shikonin induces caspase-dependent apoptotic cell death and overcomes radioresistance via the regulation of the EMT process in radio-resistant colorectal cancer cells [43]. The combination of caffeic acid phenethyl ester and paclitaxel mediates anti-tumor efficacy through the activation of cell cycle arrest and apoptosis in a paclitaxel-resistant prostate cancer model [44]. The findings of this study suggested that FAD mediates apoptosis through the PERK signaling pathway in breast cancer cell lines and that FAD can overcome radioresistance by regulating the EMT phenotype in radioresistant breast cancer cells. FAD induces caspase-dependent apoptosis through an increase in BAX and cleaved caspase-3 and a decrease in BCL2, RAB51, BRCA1, and MDC1 in hepatocellular carcinoma. In addition, FAD combined with cisplatin mediates apoptotic cell death [45].

ROS production mediates apoptotic cell death by inducing ER stress response in various cancers [46]. The natural product isoalantolactone mediates apoptosis and ER stress via the release of ROS and the downregulation of STAT3 in prostate cancer cells [47]. ROS are persistently produced by enzymatic reactions, including NADPH oxidases (NOXs), lipoxygenases, and xanthine oxidases, and then NOXs produce ROS [48]. NOX family proteins such as DUOX1, DUOX2, NOX1, NOX3, NOX4, and NOX5 are superoxide anion radical-producing enzymes that play physiological roles, including cell signaling, differentiation, gene expression, and host defense [49]. Of the NOX family proteins, NOX4 is an important source of ROS and regulates cell death and cell survival [50]. The natural compound curcumin combined with thioridazine mediates caspase-dependent apoptotic cell death by producing NOX4-induced ROS and by downregulating c-FLIP and MCL-1 in various cancers, including breast, head and neck, and glioma [51]. FAD treatment regulates ER stress-mediated apoptosis through the induction of NOX4 and the production of cytosolic ROS. Furthermore, a NOX4 knockdown combined with a DPI or NAC treatment blocked the inhibition of cell viability and the enhancement in intracellular ROS, cytotoxicity LDH, and colorimetric caspase-3 activity in FAD-induced breast cancer cell lines. NOX4 is a potential source of FAD-induced ROS generation.

Radiotherapy has been used as a common therapy to treat breast cancer [52]. However, breast cancer cells may become radioresistant. To overcome radioresistance in breast cancer patients, previous reports have indicated that combination therapies may be potential therapeutic strategies [53]. After radiotherapy, radiation exposure mediates a hypoxia environment in cancer cells and modulates the EMT phenotype [54]. During the EMT process, cell–cell contact markers, including occludin and CDH1, are decreased, and mesenchymal markers, including VIM, CDH2, and fibronectin, are increased [55]. In radioresistant MCF-7R and MDA-MB-231R cells, FAD combined with radiation inhibited the reduction of *CDH1* and the enhancement of *VIM* and *CDH2*, possibly suggesting overcoming radioresistance.

Recent studies have shown that PPARγ regulators regulate immune responses, cancer cell proliferation, lipid metabolism, and angiogenesis [56]. In both estrogen-receptor-negative and estrogen-receptor-positive breast cancer, PPARγ is a tumor suppressor [57]. The inhibitory effects of PPARγ induce apoptosis, differentiation, and cell proliferation in breast cancer [58]. The PPARγ ligands pioglitazone and rosiglitazone induce caspase-dependent apoptosis in human pancreatic cancer cells [59]. Ciglitazone induces apoptosis via the downregulation of PGE2 and COX-2 in human non-small-cell lung cancer (NSCLC) cells [60]. FAD combined with radiation overcame radioresistance through the induction of apoptosis and the UPR response and the regulation of the EMT phenotype in radio-resistant breast cancer cell lines.

## 5. Conclusions

Therefore, this study discovered the anti-breast cancer effects of FAD in vivo and in vitro. FAD mediates PPARγ activity and induces apoptotic cell death through the PERK -eIF2α-ATF4-CHOP axis, the upregulation in Nox4 expression, and the production of cytosolic Ca^2+^ and ROS in breast cancer cells. Furthermore, FAD combined with radiation overcomes radioresistance by regulating the EMT phenotype in radioresistant MCF-7R and MDA-MB-231R cells.

## Data Availability

Data are contained within the article.
